# Feasibility and Safety of 1.1‐mm Cryobiopsy for Acute Rejection Surveillance in Lung Transplant Recipients: A Comparative Study and Review of the Literature

**DOI:** 10.1155/pm/3558336

**Published:** 2026-04-15

**Authors:** Alanna Barrios-Ruiz, Alejandra Yu Lee-Mateus, Bryan F. Vaca-Cartagena, Paola Gutierrez-Gallegos, Rodrigo Funes-Ferrada, Prasanth Balasubramanian, Andras Khoor, Sadia Z. Shah, Francisco G. Alvarez, Maher Baz, Tathagat Narula, Remzi Bag, Kelly S. Robertson, Sebastian Fernandez-Bussy, David Abia-Trujillo

**Affiliations:** ^1^ Division of Pulmonary, Allergy and Sleep Medicine, Mayo Clinic, Jacksonville, Florida, USA, mayo.edu; ^2^ DeWitt Daughtry Family Department of Surgery, University of Miami Miller School of Medicine, Miami, Florida, USA, miami.edu; ^3^ Department of Internal Medicine, Clinica Redsalud Santiago; Faculty of Medicine, Universidad de los Andes, Santiago, Region Metropolitana, Chile, uandes.cl; ^4^ Department of Transplantation, Mayo Clinic, Jacksonville, Florida, USA, mayo.edu; ^5^ Department of Laboratory Medicine and Pathology, Mayo Clinic, Jacksonville, Florida, USA, mayo.edu

**Keywords:** bronchoscopy, lung biopsy, lung transplant, transbronchial cryobiopsy, transbronchial forceps

## Abstract

**Background:**

Identifying graft rejection after lung transplantation remains challenging, and no consensus exists on the optimal surveillance strategy. Transbronchial forceps biopsy (FBx) is the conventional method but is limited by small sample size and crush artifacts. Sheath‐guided cryobiopsy (CBx) using a novel 1.1‐mm probe has emerged as a promising alternative. This study evaluates the feasibility and safety of the 1.1‐mm cryoprobe and provides a focused review of current literature.

**Methods:**

We conducted a retrospective observational single‐center study between October 2022 and January 2023 of adult lung transplant recipients who underwent transbronchial biopsies for surveillance. Procedures were performed using either standard FBx or the 1.1‐mm cryoprobe. Descriptive analyses compared feasibility and safety between groups.

**Results:**

We identified 72 lung transplant recipients who underwent 109 surveillance biopsies. A total of *n* = 56 CBx and *n* = 53 FBx procedures were performed. The median procedure time was 20 min (IQR 17–26) in the CBx group versus 22 min (IQR 15–33) in the FBx group. We found a statistically higher median sample area for CBx compared with FBx (11 vs. 6 mm^2^, *p* < 0.01). No pneumothorax or postprocedural respiratory failure occurred in either group.

**Conclusion:**

CBx with a 1.1‐mm probe provides larger histological samples than FBx, with comparable safety and potentially shorter procedural times. These findings support its feasibility for lung transplant surveillance.

## 1. Introduction

Lung transplant (LTx) is the endmost life‐extending treatment for end‐stage respiratory diseases. Lung allograft transplants convey an important risk for acute cellular rejection (ACR), a host‐induced T‐lymphocyte cell‐mediated response against donor histocompatibility complex antigens within the first year of transplantation. ACR has been considered a determinant factor for chronic lung allograft dysfunction (CLAD) development, with a 5‐year survival rate (62%) inferior to other solid organ transplants [1, 2]. CLAD refers to a decline of more than 20% in forced expiratory volume in 1 s (FEV_1_%) that can manifest phenotypically as bronchiolitis obliterans (BOS), restrictive allograft syndrome (RAS), mixed, or undefined [2]. According to the International Society for Heart and Lung Transplantation (ISHLT), a global database registry of 67,493 lung transplant recipients (LTRs) pooled over 26 years, BOS remains one of the principal causes of clinical deterioration and mortality after 3–5 years of diagnosis with an increased reported incidence between 2002 and 2013 [3–5]. Therefore, optimal identification of allograft dysfunction is crucial.

Detection of organ function decline in LTR is complex. Histopathological samples and bronchoalveolar lavage (BAL) have been used as a surveillance method for identifying rejection or infection [6, 7]. The current gold standard for allograft dysfunction monitoring is transbronchial forceps biopsy (FBx). However, its sampling is limited by crush artifact caused by mechanical traction of the tip and small tissue samples, which diminish the quality and quantity of alveolar samples, potentially increasing the nondiagnostic rate [8].

Transbronchial lung cryobiopsy (CBx) has been increasingly recognized as an alternative for obtaining tissue samples for thoracic disease diagnosis [9, 10]. CBx, through the Joule–Thomson effect occurring at the distal tip of the cryoprobe, displaces gases from a space of high to low pressure, creating a rapid temperature drop that enables adjacent lung tissue to freeze, facilitating cryoextraction while preserving architectural integrity [11]. The novel 1.1‐mm cryoprobe, with an insulated catheter, provides promising advantages by increasing the bronchoscope‐free working channel area, improving flexibility, allowing 360° tissue accessibility, and facilitating maneuverability within the airway. It also potentially offers a reduced risk of crush artifacts and, unlike larger cryoprobes, does not require en bloc bronchoscope removal, which allows for maintaining a distal double‐wedging [12]. Nonetheless, bleeding safety concerns have halted its use [13]. Comparative studies of cryoprobe sizes, primarily in the context of peripheral pulmonary nodule sampling, have demonstrated that the 1.1‐mm probe yields tissue specimens of similar diagnostic quality to those obtained with larger probes (1.7‐ and 2.4‐mm) while also being associated with a lower incidence of moderate bleeding. However, these findings are based on heterogeneous populations and may not directly translate to LTR patients.

Studies comparing different probe sizes have demonstrated variation in diagnostic rate, particularly in cases of acute rejection with 2.4‐mm probe. However, despite these diagnostic advantages, the larger probe may not always be feasible in all clinical settings, especially when maneuverability or airway size is limited. Considering these findings, developing smaller, more maneuverable probes such as the 1.1‐mm cryoprobe represents a potential solution to balance diagnostic efficacy with procedural safety. To date, the performance of the 1.1‐mm cryoprobe in the LTR population remains underexplored. Here, we sought to assess the feasibility and safety of CBx with the 1.1‐mm cryoprobe compared with FBx acquisition to diagnose rejection in LTR.

## 2. Material and Methods

### 2.1. Study Participants and Design

We performed a retrospective review of single or bilateral LTRs who underwent transbronchial biopsies during their routine posttransplant care from October 1, 2022, to March 31, 2023, at Mayo Clinic Florida. This study was approved by the internal Institutional Review Board (#22‐010622). We excluded patients with the following characteristics: FEV_1_ lower than 0.8 L, severe respiratory distress, hemodynamic instability, diffuse bullous disease, coagulopathy, and a history of congestive heart failure. We analyzed and collected baseline demographic and clinical data, including age, sex, comorbidities, date, indication, and type (single or bilateral) of LTR, date of surveillance bronchoscopy and biopsy tool. All patients who underwent either CBx or FBx were included and their outcomes were analyzed per procedure, categorized by biopsy tool. Primary outcome was feasibility and safety of the 1.1‐mm cryoprobe compared with FBx. Feasibility was defined as successful completion of the planned biopsy using the assigned tool (1.1‐mm cryoprobe or forceps) without conversion to an alternative sampling technique, premature termination of the procedure, or inability to obtain tissue specimens suitable for histopathologic analysis. Safety was determined by the absence of major procedure‐related complications, including intraprocedural bleeding ≥ Grade 2 based on the Nashville Working Group Bleeding Scale [14] and postprocedure pneumothorax on chest x‐ray, hemodynamic instability, new or increased supplemental oxygen requirement due to hypoxemia, or escalation of care. Secondary outcome was evaluating diagnostic performance and sample characteristics.

### 2.2. Methods

#### 2.2.1. Bronchoscopy and Biopsy Procedure

Bronchoscopies were performed according to our center standards under general anesthesia, with deeper sedation and avoiding complete paralysis. We used an Olympus Medical Systems (Tokyo, Japan) flexible bronchoscopy with a 6.2‐mm distal end outer diameter (OD) and an instrument/working channel outlet of 2.8 mm which was advanced through a laryngeal airway mask. Patients first underwent prebiopsy BAL, using 100 cc of standard saline solution. The targeted lobe was selected prior to intervention based on the transplanted lobe location and previous chest imaging. All procedures were performed by four LTx surgeons and two interventional pulmonologists.

We acquired tissue from different segments and lobes; the number of obtained samples was at the discretion of each proceduralist. The choice to perform either CBx or FBx depended on proceduralist expertise and preference, and only one biopsy tool was used per procedure. Forceps tissue samples were obtained with a standard disposable 2.2‐mm radial Jaw 4 forceps for FBx with an OD of 1.8 mm (Boston Scientific, United States) and with a 1.1‐mm OD single use over sheath cryoprobe for CBx (ERBE, Germany). FBx was performed in standard fashion. If CBx was selected, the procedure was as follows: A therapeutic bronchoscope advanced into the airway until wedge position was achieved. The over sheath was inserted through the bronchoscope instrument channel and locked in a steady position. The probe was introduced, under fluoroscopic guidance, until a slight resistance was felt, and the visceral pleura was reached. Subsequently, we retracted the scope from 1 to 2 cm under fluoroscopic visualization. Freezing times ranging from 3 to 6 s were applied subject to tissue resistance and biopsy specimen size. Only the probe was removed within each tissue collection, whereas the freezing pedal remained activated upon retraction, leaving the bronchoscope and over sheath in wedge position.

#### 2.2.2. Histology

Specimens were processed and stained with hematoxylin and eosin using routine histological methods. No immunohistochemistry or special stains were applied. Pathology evaluation included assessment for ACR, airway inflammation, and quantification of alveolated parenchyma and airway wall presence in each sample. ACR was graded according to the ISHLT guidelines: Grade A (A0–none, A1–minimal, A2–mild, A3–moderate, and A4–severe); airway inflammation and lymphocytic bronchiolitis as Grade B (B0–none, B1–low grade, B2–high grade, and BX–ungradable); and chronic rejection, obliterative bronchiolitis, as Grade C (C0–absent and C1–present) [15].

Alveolated parenchyma surface area was measured using image analysis software (SECTRA, Linköping, Sweden), focusing exclusively on regions of preserved alveolar tissue. Areas with hemorrhage or crush artifacts were excluded to ensure accurate measurement of viable alveolar surface. Although pathologists were not blinded to the biopsy modality, multiple observers performed initial evaluations, and a pulmonary pathologist (A.K.) made the final confirmation, aiming to reduce interobserver variability and increase diagnostic consistency.

### 2.3. Complications

Procedural complications were noted intraprocedure and postprocedure, including hemodynamic instability, increased or new supplemental oxygen requirements for hypoxemia, and escalation of care. Endobronchial intraprocedural bleeding was classified according to the Nashville Working Group Bleeding scale, with a score of 2 or more. A chest x‐ray was obtained after each procedure for pneumothorax assessment.

### 2.4. Radiation

Two different mobile C‐arm fluoroscopes were used for navigation. When available, we collected procedural data on milligrays (mGy), square milligrays (mGy^2^), and fluoroscopy time.

### 2.5. Review of Literature

#### 2.5.1. Search Strategy

A literature review was conducted using PubMed, Medline (OVID), and Scopus to identify studies that evaluated CBx and/or transbronchial forceps in LTRs. We limited our search to articles between January 2013 and June 2025, in English and Spanish languages. The search terms used were “Cryobiopsy,” “Cryoprobe biopsy,” “Transbronchial lung cryobiopsy,” “Transbronchial cryobiopsy,” “Lung transplant patients,” “Lung transplant recipients,” “Lung transplantation,” “Lung allograft recipients,” or “Acute cellular rejection.” Our initial search retrieved 121 articles; after duplicate removal, 70 articles were screened by abstract and title. We excluded case reports, review articles, and two studies which included fewer than five patients. One additional study was included using the snowballing method. This revision includes the findings of 11 relevant studies.

### 2.6. Statistical Analysis

Data were summarized using descriptive statistics. Distribution of data was assessed with visual plots and the Shapiro–Wilk test. Continuous variables that were not normally distributed were presented as medians with interquartile ranges (IQR), whereas normally distributed variables were reported as means with standard deviations. Categorical variables are presented as counts and percentages. Associations between variables were assessed using Fisher exact test. All analyses were conducted using R Statistical Software Version 4.4.2. [16]

## 3. Results

A total of 72 LTx recipients underwent 109 surveillance bronchoscopies. The median age was 63 years (IQR, 54–69), 48 (67%) were male, and the median BMI was 27 kg/m^2^ (IQR, 24–29). Fifty‐nine (82%) patients had bilateral transplants; of these, four (7%) patients had a history of a repeat transplant intervention due to CLAD and one due to primary graft dysfunction (2%). Most common indications for LTx were pulmonary fibrosis (33%), interstitial lung disease including the spectrum of (UIP, IIP, and NSIP) (21%), chronic obstructive pulmonary disease (COPD) (21%), severe acute respiratory syndrome coronavirus‐2 infection (13%), bronchiectasis (6%), cystic fibrosis 3 (4%), and others (2%). Baseline characteristics are listed in Table 1.

**Table 1:** Demographic and clinical characteristics.

Variable	*O* *v* *e* *r* *a* *l* *l* *n* = 109	*C* *B* *n* = 56	*F* *B* *x* *n* = 53	*p*value
Sex (%)				
Female	34 (31.2)	19 (33.9)	15 (28.3)	0.543
Age (IQR)	63 (54–69)	63.5 (52–68.2)	63 (55–69)	0.718
BMI (kg/m^2^) (SD)	27 (4.3)	26.5 (4)	27.6 (4.6)	0.203
Race (%)				0.630
White	86 (78.9)	46 (82.1)	41 (77.4)	
African American	10 (9.2)	6 (10.6)	4 (7.4)	
Asian, Filipino	2 (1.8)	1 (1.7)	1 (1.8)	
Asian, Vietnamese	1 (0.9)	0 (0)	1 (1.8)	
Other	9 (8.3)	3 (5.3)	6 (11.3)	
Ethnicity (%)				0.549
Hispanic or Latino	12 (97)	5 (8.93)	7 (13.2)	
Not Hispanic or Latino	11 (89)	51 (91.1)	46 (86.8)	
Smoking history (%)				0.697
Former	54 (48.5)	29 (51.7)	25 (47.2)	
Never	52 (49.1)	25 (44.6)	27 (50.9)	
NA	3 (2.4)	2 (3.57)	1 (1.8)	
Comorbidities (%)				
Hypertension	80 (73.4)	42 (75)	38 (71.7)	0.830
Diabetes mellitus	58 (53.2)	29 (51.8)	29 (54.7)	0.703
CKD	47 (43.1)	23 (41.1)	24 (45.3)	0.698
COPD	27 (24.8)	15 (26.8)	12 (22.6)	0.824
Type of first transplant				
Bilateral	85 (78)	42 (75)	43 (81.1)	0.509
Left	13 (11.9)	7 (12.5)	6 (11.3)	1
Right	11 (10.1)	7 (12.5)	4 (7.4)	0.478
Site of biopsy				
RUL	18 (16.5)	5 (8.8)	13 (24.5)	0.031
RML	13 (11.9)	7 (12.5)	6 (11.2)	1
RLL	53 (48.6)	33 (58.9)	20 (37.7)	0.077
LUL	6 (5.5)	1 (1.7)	5 (9.3)	0.209
LLL	19 (17.4)	10 (17.9)	9 (17)	1

Abbreviations: CKD, chronic kidney disease; COPD, chronic obstructive pulmonary disease; IQR, interquartile range; LLL, left lower lobe; LUL, left upper lobe; RLL, right lower lobe; RML, right middle lobe; RUL, right upper lobe.

### 3.1. Bronchoscopy

One hundred and eight surveillance bronchoscopies were performed in 72 recipients. More than one procedure was performed in 28 recipients: two bronchoscopies in 21, three in 5, and four in 2 recipients. Biopsy sites were right lower lobe 56 (52%), right middle lobe 25 (23%), left lower lobe 19 (17%), left upper lobe 7 (6%), right upper lobe 2 (2%) (Table 2).

**Table 2:** Procedure characteristics.

Variables	Total procedures ✝ (*N* = 109)	CBx (*N* = 56)	FBx (*N* = 53)	*p* value
Length of procedure, min	22 (16–30)	20 (17–26)	22 (15–33)	0.692
Number of specimens	6 (5–8)	5 (4–6)	7 (5–8)	**0.001**
Sample area, mm^2^	9 (4–17)	11 (7–20)	6 (3–14)	**0.001**
Specimens containing lung parenchyma	5 (4–6)	5 (4–6)	5 (4–8)	0.097
Specimens containing airway wall	2 (1–3)	2 (1–2)	3 (1–3)	**0.001**

*Note:* ✝denotes patients who underwent repeated surveillance bronchoscopies; therefore, the number of procedures is greater than the number of individual patients. All values include median and IQR. Boldface data refers to *p* values representing a statistical difference, as defined by a *p* value < 0.05.

We categorized procedures by biopsy tool used: 56 (51%) in the CBx group and 53 (49%) in the FBx group (Table 2). For CBx, the median time of procedure was 20 min (IQR 17–26), the median number of evaluable biopsy specimens was 5 (IQR 4–6), with a median of 11 mm (IQR 7–20) in sample area. For FBx, the median time of procedure was 22 min (IQR 15–33), the median number of evaluable biopsy specimens was 7 (IQR 5–8), with a median of 9 mm^2^ (IQR 4–17) in sample area. Fluoroscopy and radiation data were available in CBx (*n* = 40) and FBx (*n* = 22) procedures. Reported median fluoroscopy time was 2.5 min (IQR 1.4–3.5) and median mGy and mGy^2^ doses were 37.9 mGy (IQR, 27.3–70.5) and 443.2 mGy^2^ (IQR, 272.4–807.7), respectively, for CBx, compared with 4.5 min (IQR 3.5–5.2), 113.2 mGy (IQR, 68–143.5), 1543.8 mGy^2^ (IQR, 938.5–1909.8) for FBx, all statistically greater for FBx (*p* < 0.001).

In the Nashville Bleeding scale, only 4 (3.7%) procedures, 2 within each group, had a score of 2, meaning that there was a need of suctioning for more than 1 min or other therapeutic bronchoscopic intervention [15]. No other postprocedure complications, including severe bleeding, pneumothorax, respiratory failure, or death, were reported.

### 3.2. Biopsy Samples and Histopathology

When comparing the sample details, we found a significantly higher number of specimens collected for FBx (7 vs. 5; *p* = 0.001) and a larger median sample area for CBx (11 mm^2^ vs. 6 mm^2^; *p* = 0.001) (Table 2).

**Figure 1:**
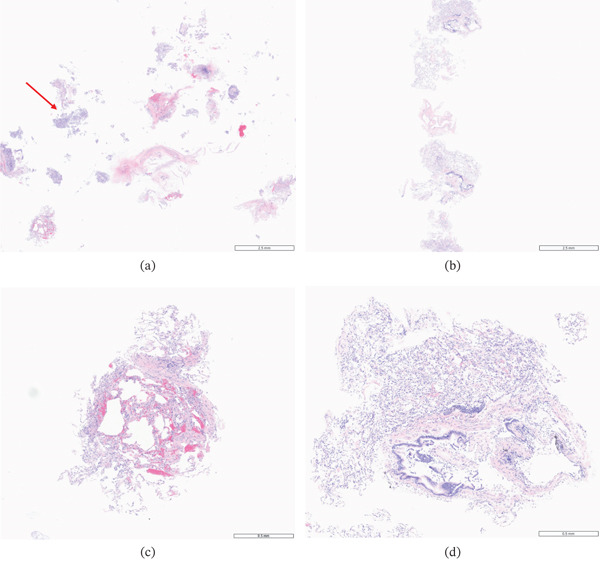
Histological images of postlung transplant transbronchial biopsy specimens obtained by forceps and cryobiopsy with the 1.1‐mm cryoprobe of the same patient. (a, c) Histological images of forceps biopsy at 8× and 40× magnifications in a 2.5‐ and 5‐mm scale bar, respectively, in hematoxylin and eosin stains. Forceps biopsy shows crush artifact (red arrow). (b, d) Histological images showing cryobiopsy at 8× and 40× magnifications in a 2.5‐ and 5‐mm scale bar, respectively, in hematoxylin and eosin stains. Cryobiopsy samples present larger tissue surface area. Both specimens show no rejection (Grade A0).

Of the 109 specimens, 93 (86%) cases scored A0, 11 (11%) A1, 2 (2%) A2, 1 (1%) A3, 45 (42%) B0, 56 (51%) Bx, and 27 (25%) C0, according to the classification and grading of pulmonary allograft rejection. CBx and FBx (Figure 1) showed comparable results in their diagnostic performance, as shown in Table 3.

**Table 3:** Histopathological findings according to ISHLT criteria.

Classification	Frequency, (%) (*N* = 109)	CBx (*N* = 56)	FBx (*N* = 53)	*p*value
A0	93 (86)	49 (87)	45 (85)	0.683
A1	11 (11)	5 (8)	6 (10)	0.888
A2	2 (2)	1 (2)	1 (1)	0.967
A3	1 (1)	0 (0)	1 (1)	—
B0	45 (42)	22 (39)	23 (44)	0.737
C0	27 (25)	16 (28)	11 (19)	0.599
Bx	56 (51)	27 (48)	29 (51)	0.824

*Note:* Acute cellular rejection was classified as follows: acute rejection as Grade A: A0–none, A1–minimal, A2–mild, A3–moderate, and A4–severe; airway inflammation and lymphocytic bronchiolitis as Grade B: B0–none, B1–low grade, B2–high grade, and BX–ungradable; and chronic rejection, obliterative bronchiolitis as Grade C: C1–present or C0–absent.

Abbreviation: ISHLT, International Society for Heart and Lung Transplantation.

## 4. Discussion

LTR management remains clinically challenging, especially when diagnosing allograft dysfunction early and accurately. Functional tests such as spirometry, BAL, or imaging have limited sensitivity, whereas newer biomarkers like extracellular vesicles or free‐circulating nucleic acids face technological and clinical adoption hurdles [17–19]. Histologic sampling through transbronchial biopsy remains the diagnostic gold standard for ACR, and optimizing biopsy techniques is crucial for improving care. A review of literature on transbronchial biopsy for detecting ACR is found in Tables 4 and S1.

**Table 4:** Overview of cryobiopsy and/or forceps biopsy within current literature on lung transplant.

First Author	No. Patients	Type of Biopsy Tool (No.)	Probe Size (mm)	Procedural Bleeding Moderate to Severe	Pneumothorax, Pleural Drainage	Diagnostic Samples (Definitive, Probable)	Non‐diagnostic	Relevant findings
Balasubramanian 2025 [20]	49	FBx + CBx (49)	1.1	3	0,0	47, 49	2, 0	CBx with the 1.1mm cryoprobe provided higher diagnostic yield than FBx in the same patient.
Steinack 2025 [21]	80	FBx + CBx (40)	2.4	15	1,0	86	4	Diagnostic yield for CBx + FBx and CBx only, 95.6% vs 97.7%, the sole use of CBx alone did not lead to a lower ACR incidence compared to the combined group RR = 2.21, 95% CI 0.67‐7.29; P=0.190).
		CBx (40)				85	2	
Thiboutot 2022 [12]	25	CBx (25)	1.1	0	2,0	23	2	Satisfactory safety profile with 1.1mm CBx probe.
Steinack 2022 [7]	35	FBx	**-**	23	4,4	6	57	Moderate interobserver agreement between pathologists FBx vs CBx (KI = 0.54).
		CBx	2.4,1.7	1		60	3	
Mohamed 2020 [22]	54	FBx (223)	**-**	3	7,7	**-**	**-**	Diagnostic rate of acute rejection using TCB was 100% compared with 83% using conventional FBx (P<.001).
	110	CBx (75)	**-**	6	1,1	**-**	**-**	
Loor 2019 [23]	206	CBx (321)	2.4	24	25,12	257,60	4	Histological diagnostic certainty increases with 4 tissue samples (P < .001).
Montero 2018 [24]	81	FBx (41)	Alligator Jaw Step	6	0,0	26,6	9	Larger samples (P<0.0001), absence of crush artifacts (P=0.002), and a diagnostic yield for acute (P=0.0657) and chronic (P=0.0053) cellular rejection with CBx.
		CBx (40)	2.4	9	5,3	28,11	1	
Gershman 2018 [25]	362	FBx (201)	1.9 Fenestrated alligator	4	8, ‐	197	4	CBx safety with lower rate of crush artifacts (P<.001).
		CBx (201)	2.4	5	9, ‐	199	2	
Roden 2014 [26]	18	CBx (27)	1.9	0	1, ‐	‐	‐	CBx larger size, less crush artifact (p<.001) with frequent complications. No significant difference between reviewers’ agreement on biopsy grading.
Fruchter 2013 [27]	40	FBx (40)	Radial Jaw 3	3	1, 0	37	3	Short fluoroscopy time in CBx vs FBx [25 s vs 90 s] (P < 0.05).
	40	CBx (40)	2.4	1	0, 0	40	0	
Yarmus 2013 [28]	17	FBx	‐	1	1, ‐	‐	‐	Larger specimen area and percent of open alveoli with CBx (P < .05).
		CBx	1.8	0	1, ‐	‐	‐	

CBx: Cryobiopsy, FBx: Forceps biopsy.

### 4.1. Biopsy Protocol, Freezing Time, and Sample Size

The ability to retrieve adequate lung tissue during transbronchial biopsy is essential for a reliable histological diagnosis. According to the ISHLT guidelines, at least five pieces of alveolated lung parenchyma, ideally with at least one or two bronchiolar structures, are recommended for accurate interpretation of lung allograft pathology [23, 29, 30]. In our cohort, although the number of samples obtained was lower in the CBx group than in the FBx group (median 5 vs. 7, *p* < 0.001), the median alveolated parenchymal area was significantly greater in CBx specimens (11 vs. 6 mm^2^, *p* < 0.01).

These findings align with previous reports that have shown that CBx yields larger samples than FBx. For example, Montero et al. reported an average area of 22.1 mm^2^ versus 8.5 mm^2^ with CBx and FBx, respectively, whereas Roden et al. found a mean area of 50 mm^2^ using a 1.9‐mm cryoprobe [24, 26]. Our measured values were smaller, likely due to the 1.1‐mm probe size and the strict inclusion of only intact alveolated parenchyma in our image analysis. Hemorrhagic and artifact‐damaged areas were intentionally excluded to ensure that only viable, interpretable lung parenchyma was quantified. This methodological difference likely explains the lower sample area relative to other studies, such as Thiboutot et al. who reported a mean CBx sample area of 54.4 mm^2^ using the same 1.1‐mm probe [12]. Nevertheless, a prior institutional study comparing CBx with FBx in a different cohort reported a median alveolated tissue area of 19.3 mm^2^ for CBx compared with 5.4 mm^2^ for FBx as well as Steinack et al. who described median sample sizes of 7 mm^2^ and 2 mm^2^ for CBx and FBx alone, respectively [20, 21]. Despite the overall smaller tissue samples, CBx remained significantly greater in size than FBx in the evaluated cohort in both studies (*p* < 0.001).

There is currently no standardized CBx protocol for LTRs, especially regarding ideal freezing time and number of samples. Literature shows wide variability in technique, with freezing times ranging from 3 to 7 s and biopsy counts ranging from 2 to 7 per procedure. In our study, freezing time was tailored per case based on tissue resistance and proceduralist judgment. This variability, combined with proceduralist‐dependent sampling patterns, may introduce confounding and underscores the need for protocol harmonization.

Importantly, although fewer samples were obtained with CBx, a comparable number of evaluable specimens containing alveolated parenchyma were obtained in both groups, supporting equivalent or potentially enhanced tissue efficiency for CBx. However, the operator‐driven sampling strategy likely influenced both sample quantity and quality. Further controlled studies are necessary to determine whether increasing the number of CBx passes will improve diagnostic performance or if the inherent greater tissue volume and efficiency can compensate for fewer samples.

### 4.2. Diagnostic Samples and Specimen Quality

Although FBx yielded a higher median number of specimens per procedure (7 vs. 5, *p* < 0.001), the proportion of specimens containing alveolated parenchyma was comparable between groups. Diagnostic adequacy remained high across both modalities. Notably, a higher percentage of CBx samples contained intact alveolated tissue with fewer crush artifacts, a finding supported by prior studies [24–28]. In a recent randomized controlled trial comparing CBx alone with CBx combined with FBx to identify ACR, CBx alone did not detect lower ACR than the combined group, with similar diagnostic yields and a larger tissue sample size [21]. Although the number of specimens was not reported, CBx alone showed an optimal overall performance.

There are certain challenges while assessing diagnostic value in a population like ours, as the ACR diagnosed here was mainly a surveillance procedure of asymptomatic patients while on immunosuppressive regimens [19]. Most of the literature has focused on asymptomatic recipients undergoing protocol or surveillance biopsies. Furthermore, the high degree of variability and low reproducibility of the histopathologic sample′s interpretation of ACR (kappa value for interobserver agreement of 0.183 [31] and 0.54 [7] contributes to the complexity of extrapolating the reduction in artifact rates and its diagnostic impact during sample interpretation).

The number of evaluable specimens (defined here as samples containing ≥ 1 mm^2^ of preserved alveolated tissue) differed between groups and may reflect proceduralist preferences or intraprocedural judgment. Given that FBx often retrieves smaller but more numerous samples, future protocols should consider both quality and quantity of tissue, especially in histologically heterogeneous diseases like rejection.

The presence of airway wall in biopsy specimens, also reported in our study, remains of unclear diagnostic value. Although ISHLT guidelines recommend obtaining at least 1–2 samples containing bronchioles, the relevance of this parameter has not been consistently validated and was not associated with differences in outcomes in our cohort.

### 4.3. Procedural Time, Fluoroscopy, and Safety

CBx procedures were associated with shorter fluoroscopy times (2.5 vs. 4.5 min, *p* < 0.001), though procedure duration was similar. These results are consistent with prior studies^24^ but must be interpreted in context: CBx patients often received fewer total biopsies, and proceduralist preferences likely contributed to this difference.

Importantly, no major complications were observed in either group. Prior studies have reported pneumothorax and moderate bleeding at low but nonzero rates [12]. Although Balasubramanian et al. reported two three instances of Nashville Scale 2 bleeding requiring suctioning for more than 1 min, repeat wedging, or instillation of hemostatic agents, both biopsy tools were used in each patient, which may have contributed to an increased risk of complications. The 1.1‐mm cryoprobe appears to have a favorable safety profile, avoiding the need for en bloc removal and maintaining airway wedging. This may reduce bleeding risks seen with larger probes. Nevertheless, the sample size limits conclusions about rare complications.

### 4.4. Immunohistochemistry Staining and Molecular Test to Diagnose Rejection

Routine staining for lung allograft involves the use of hematoxylin–eosin, Grocott methenamine silver, Verhoeff–Van Gieson, periodic acid–Schiff and/or Movat stain. Although these methods remain standard, the emergence of humoral rejection markers has been proven to support the diagnosis of antibody‐mediated rejection in kidney and heart transplants [32]. This form of immune dysfunction, though less frequently identified in LTxs, has been implicated in the progression to CLAD [26, 33]. However, the utility of endothelial capillary markers such as diffuse C4d remains controversial due to their low sensitivity, inconsistent findings, and the confounding role of donor‐specific antibodies [29].

In addition, there are two methods for immunopathological assay detection of C4d, frozen‐tissue immunofluorescence and formalin‐fixed paraffin immunohistochemistry, but intermethod discrepancies have been reported, particularly in cardiac transplantation [34]. Among the studies reviewed in our analysis, only two reported the use of special stains in LTx biopsies. This highlights a gap in current practice and underscores the need for further research comparing histochemical protocols. Standardized approaches, such as the LASHA framework, may offer greater consistency in biopsy interpretation and could contribute to improved diagnostic yield in future studies [30].

### 4.5. BAL in Posttransplant Surveillance

Although histopathology remains the diagnostic gold standard for ACR, BAL can provide a representative sample of the bronchoalveolar allograft microenvironment and may offer complementary prognostic information [35]. In our study, BAL was obtained prior to biopsy in all procedures and was intended to assess for infectious and inflammatory parameters, not for detection of ACR per se. Emerging evidence has demonstrated that specific BAL cytokines, including CXCL10, IL6, S100A8, pentraxin‐3, TNF‐receptor‐1, and IL10, are associated with subsequent CLAD development and/or death, even in clinically stable patients or those with indeterminate biopsy findings [36]. Earlier work has similarly shown that BAL inflammatory markers obtained during surveillance bronchoscopies can identify patients at increased risk for adverse long‐term outcomes independent of ACR grade. Although not incorporated into our outcomes, this recent data supports the role of BAL as a complementary tool for risk stratification and for improving non‐invasive surveillance approaches [37].

### 4.6. Strengths and Limitations of the Study

Strengths of the study include its applicability in real‐world clinical settings and the incorporation of systematic histological assessment through image‐based analysis and separate assessments for CBx and FBx as standalone diagnostic tools. Nonetheless, several limitations must be acknowledged. Some patients underwent more than one bronchoscopy with different biopsy modalities; each procedure was treated as an independent intervention for the purposes of statistical analysis. The choice of biopsy modality was not randomized but rather determined by individual proceduralist preference per procedure, which may have introduced both selection and performance bias. Additionally, the involvement of operators with varying levels of experience could have affected procedural outcomes such as fluoroscopy duration and the number of samples obtained. The absence of blinding among proceduralists and pathologists may have introduced interpretive bias, as prior knowledge of the biopsy modality and its expected tissue characteristics could have led to underinterpretation or overinterpretation of the specimens. A favorable safety profile was described for CBx; however, the relatively small sample size limits the statistical power to detect rare complications. Finally, due to the nonrandomized and retrospective nature of our study, our findings should be interpreted as feasibility and safety of the 1.1‐mm cryoprobe for detection of ACR, showing improved tissue efficiency rather than definitive evidence of superior diagnostic accuracy. Although CBx provided larger samples with preserved architecture, our study was not powered nor designed to demonstrate improved diagnostic sensitivity for clinically significant rejection.

## 5. Conclusion

CBx with a 1.1‐mm probe demonstrated feasibility and safety in this cohort of LTRs. Compared with FBx, CBx yielded larger alveolated tissue samples, which are essential for rejection assessment, with fewer biopsy passes, shorter fluoroscopy time, and no increase in complications. Large‐scale, prospective randomized controlled studies are warranted to validate these findings and define the role of CBx in posttransplant surveillance.

**Nomenclature**
ACR: acute cellular rejection
CBx: cryobiopsy
CLAD: chronic lung allograft dysfunction
FBx: forceps biopsy
LTx: lung transplant
LTR: lung transplant rejection

## Author Contributions

A.B‐R. was involved in writing the original draft, review and editing, and data curation. A.Y.L‐M., B.F.V‐C., P.G‐G., and R.F‐F. were involved in review, editing, and data curation. P.S. was involved in writing the original draft, review, editing, and formal analysis. A.K., F.G.A., M.B., T.N., S.Z.S., R.B., and K.S.R. were involved in supervision and methodology. S.F‐B. and D.A‐T. were responsible for conceptualization, investigation, methodology, and writing the original draft, review, and editing.

## Funding

No funding was received for this manuscript.

## Disclosure

Part of this manuscript was accepted as an abstract for poster presentation at the ISHLT 44th Annual Meeting and Scientific Sessions Prague Congress held in Centre Prague, Czech Republic from April 10 to 13, 2024.

## Conflicts of Interest

The authors declare no conflicts of interest.

## Supporting information


**Supporting Information** Additional supporting information can be found online in the Supporting Information section. Additional supporting information can be found online in the Supporting Information section. Table S1: Baseline characteristics and procedural and histopathological details of studies assessed in the literature review on acute rejection surveillance in lung transplant recipients. ∗CBx: cryobiopsy, FBx: forceps biopsy, RCT: randomized controlled trial. ∗Steinack et al.′s study reported median sample size of 7 mm (5–10) for the combined CBx and FBx group and a separate median sample size of 2 mm (1–3) for FBx alone.

## Data Availability

The data that support the findings of this study are available on request from the corresponding author. The data are not publicly available due to privacy or ethical restrictions.
