# Supplementary material for: Feasibility and Safety of 1.1‐mm Cryobiopsy for Acute Rejection Surveillance in Lung Transplant Recipients: A Comparative Study and Review of the Literature
Source: Pulm Med. 2026 Apr 15;2026:3558336. doi: 10.1155/pm/3558336 (PMC13080343; doi:10.1155/pm/3558336)
Supplement: Supplementary file 1 — Supporting Information Additional supporting information can be found online in the Supporting Information section. Additional supporting information can be found online in the Supporting Information section. Table S1: Baseline characteristics and procedural and histopathological details of studies assessed in the literature review on acute rejection surveillance in lung transplant recipients. ∗CBx: cryobiopsy, FBx: forceps biopsy, RCT: randomized controlled trial. ∗Steinack et al.′s study reported median sample size of 7 mm (5–10) for the combined CBx and FBx group and a separate median sample size of 2 mm (1–3) for FBx alone. [file PM-2026-3558336-s001.docx]

**Supplementary Table 1.** Table 1. Baseline characteristics and procedural and histopathological details of studies assessed in the literature review on acute rejection surveillance in lung transplant recipients

*CBx: Cryobiopsy, FBx: Forceps, RCT: Randomized Controlled Trial*

*** Steinack et al. study reported median sample size of 7mm (5-10) for the combined CBx and FBx group and a separate median sample size of 2mm (1-3) for FBx alone.

| **#** | **First Author** | **Study Design** | **Country** | **Type of Procedure** | **Asymptomatic Recipients** | **Biopsy Protocol ( ± freezing time seconds)** | **Total average area of sample (mm²)** | **Biopsies with crush artifacts** | **Special Staining** |
| --- | --- | --- | --- | --- | --- | --- | --- | --- | --- |
| 1 | Balasubramanian (2025) | Prospective | USA | FBx    CBx | 49 | 6 specimens  5 specimens | 5.4 (2.4-9.1)  19.3 (12.3-24.9) | **-**  **-** | **-**  **-** |
| 2 | Steinack (2025) | RCT | Switzerland | CBx  CBx + FBx | 40  40 | -  - | 7 (6-10)  7 (5-10)  2 (1-3)* | **-**  **-** | **-**  **-** |
| 3 | Thiboutot (2022) | Prospective | USA | CBx | 8 | Minimum of five specimens (4) | 54.4 ± 33.3 | **-** | **-** |
| 4 | Steinack (2022) | Prospective, sequential procedures | Switzerland | CBx | 46 Procedures | 5 specimens by FB, 2 by CB (4 to 5 s with 2.4 mm CB or 6 to 7 s with 1.7 mm CB) | **-** | **-** | **-** |
|  |  |  |  | FBx |  |  | **-** | **-** |  |
| 5 | Mohamed (2020) | Retrospective | Italy | FBx | 54 | **-** | **-** | **-** | **-** |
|  |  |  |  | CBx | **-** | **-** | **-** | - |  |
| 6 | Loor (2019) | Prospective | Spain | CBx | 60 | 3-6 samples for different segments of the lobe | 84.27 (8-289) | 7 | **-** |
| 7 | Montero (2018) | Prospective | Spain | FBx | - | 6 samples from different segments of the lobe (3) | 8.5 ± 6.5 (40–1.5) | 9 | C4d |
|  |  |  |  | CBx |  |  | 22.1 ± 12.5 (56.2–4.8) | 0 |  |
| 8 | Gershman (2018) | Retrospective | Israel | FBx | 362 | 4-6 samples | 6.6 | 11 |  |
|  |  |  |  | CBx |  | 2-3 specimens (4) | 16.6 | 0 | **-** |
| 9 | Roden (2015) | Retrospective | Rochester | CBx | 10 | 3 (3-5) | **-** | 8 | C4d |
| 10 | Fruchter (2013) | Retrospective | Israel | FBx | 27 | 7 (6-8) | 2 | 0 | **-** |
|  |  |  |  | CBx | 26 | 2-3 biopsies taken from the same place, cooled approx. (4) | 10 | 0 |  |
| 11 | Yarmus (2013) | Prospective | USA | FBx | 17 | 10 samples | 12.5 | 21 | **-** |
|  |  |  |  | CBx |  | 5 samples (3) | 50 | 0 |  |
